# The Behavior and Mind Health (BeMIND) study: Methods, design and baseline sample characteristics of a cohort study among adolescents and young adults

**DOI:** 10.1002/mpr.1804

**Published:** 2019-12-05

**Authors:** Katja Beesdo‐Baum, Catharina Voss, John Venz, Jana Hoyer, Johanna Berwanger, Hanna Kische, Theresa Magdalena Ollmann, Lars Pieper

**Affiliations:** ^1^ Behavioral Epidemiology, Institute of Clinical Psychology and Psychotherapy Technische Universität Dresden Dresden Germany; ^2^ Center for Clinical Epidemiology and Longitudinal Studies (CELOS), Institute of Clinical Psychology and Psychotherapy Technische Universität Dresden Dresden Germany

**Keywords:** cohort study, epidemiology, etiology, health behavior, psychopathology

## Abstract

**Objectives:**

The Behavior and Mind Health (BeMIND) study is a population‐based cohort study of adolescents and young adults from Dresden, Germany. The aim is to investigate psychological and behavioral factors linked to a range of mental disorders and health behaviors and their interaction with social‐environmental and genetic/biologic factors.

**Methods:**

A random sample of 14–21 year olds was drawn from the population registry in 2015. The baseline investigation was completed 11/2015–12/2016 (*N* = 1,180). Assessments include standardized diagnostic interview, cognitive‐affective tasks, questionnaires, biosamples, and ecologic momentary assessment in real life with combined actigraphic/geographic monitoring. In the family study component, parents completed similar assessments and provided information on child's early development.

**Results:**

The participation rate (minimum response proportion) was 21.7%; the cooperation rate was 43.4%. Acceptance and completion of study components were high. General health data indicate that more than 80% reported no or only mild impairment due to mental or somatic health problems in the past year; about 20% ever sought treatment for mental health problems or chronic somatic illnesses, respectively.

**Conclusions:**

Data from BeMIND baseline and follow‐up investigations will provide novel insights into contributors to health and disease as adolescents grow into adulthood.

## 1 INTRODUCTION

Mental and behavioral disorders have been shown to be jointly responsible for the largest proportion of disability burden worldwide (Erskine et al., 2015; Kessler et al., 2011; Whiteford, Ferrari, Degenhardt, Feigin, & Vos, 2015; Wittchen et al., 2011). A wealth of epidemiological data shows a high prevalence in the general population (Beesdo‐Baum & Wittchen, 2015; Polanczyk, Salum, Sugaya, Caye, & Rohde, 2015), a typically early onset in youth, and frequent persistence into adulthood (Beesdo, Knappe, & Pine, 2009; Copeland et al., 2013; Kessler et al., 2005). It is also well established that mental and behavioral disorders result from complex vulnerability and risk factor constellations including family‐genetic, individual, environmental, and societal factors (Beesdo‐Baum et al., 2015; Wille, Bettge, & Ravens‐Sieberer, 2008), whereas protective factors may promote mental health or buffer the adverse effects of risk factors (resiliency; Sapienza & Masten, 2011). Initial disorders (e.g., anxiety disorders) have been shown to increase themselves the risk for the temporally secondary onset of other psychopathological conditions (e.g., depression) and even somatic diseases (e.g., cardiovascular disease; Beesdo et al., 2007; Copeland et al., 2013; De Hert, Detraux, & Vancampfort, 2018; Wittchen et al., 2007), indicating the potential usefulness of staging‐ or symptom‐progression models for improved classification and targeting interventions (McGorry, 2007; Wittchen et al., 2014). However, the interplay between contributing factors and particularly the trajectories and mechanisms of symptom development and progression remain far from conclusive (Forsman et al., 2015; Verhulst & Tiemeier, 2015; Wittchen, Knappe, & Schumann, 2014). This likely constitutes the basis for previous prevention efforts resulting in only small effects, particularly if applied universally (obesity: Bleich, Segal, Wu, Wilson, & Wang, 2013; depression: Calear & Christensen, 2010; anxiety: Fisak, Richard, & Mann, 2011; behavior disorders: Hautmann, Hanisch, Mayer, Plurck, & Dopfner, 2008; substance use: Lemstra et al., 2010). Selective or indicative preventive programs revealed more promising results (Ginsburg, Drake, Tein, Teetsel, & Riddle, 2015; Lau & Rapee, 2011; Stice, Shaw, Bohon, Marti, & Rohde, 2009), suggesting to tailor interventions to the individual needs of a person (Hamburg & Collins, 2010; Jain, 2009).

Although behavioral factors, as defined within a larger psychological or behavioral science perspective, are deemed a core contributor to almost all ill‐health conditions, their consideration and objective assessment in epidemiological studies have so far been limited (Wittchen, Knappe, Andersson, et al., 2014). Improved knowledge on the behavioral and psychological determinants, including cognitive‐affective factors and decision‐making processes, in the evolution of mental disorders and health risk behaviors contributing to somatic disease, and the interplay of these factors with genetic/biological and environmental factors, may improve etiopathogenetic models and targeted interventions suitable for changing disease trajectories.

We therefore launched a prospective‐longitudinal epidemiological study focusing on mental disorders and health risk behaviors in adolescents and young adults, in which traditional subjective, retrospective assessments of mental and behavioral health and disorders, as well as a range of individual, familial, and social‐environmental risk/protective factors, are complemented by more ecologically valid and objective measures of subjects' health and behavior in real life and in controlled (experimental‐laboratory) environments. The study's overarching aim is to contribute to an improved understanding of the functional and dysfunctional psychological and behavioral factors and processes and their interaction with genetic/biological and environmental factors in the maintenance of health and the critical trajectories into mental disorders and health risk behaviors linked to noncommunicable somatic disease. Specific objectives are (a) to assess mental disorders and health behaviors in a population‐based sample of adolescents and young adults both cross‐sectionally and longitudinally; (b) to monitor changes in mental health symptoms and health behaviors, both on a microlevel in daily life using ecological momentary assessment and prospectively from baseline to one and 3‐year follow‐up; (c) to identify etiological pathways considering distal and proximal individual (psychological/behavioral) risk and protective factors as well as their interactions with social‐environmental and biologic/genetic factors; and (d) to identify predictors for changes in mental health status and health behaviors.

## 2 METHODS

### 2.1 Study design

**Figure 1:**
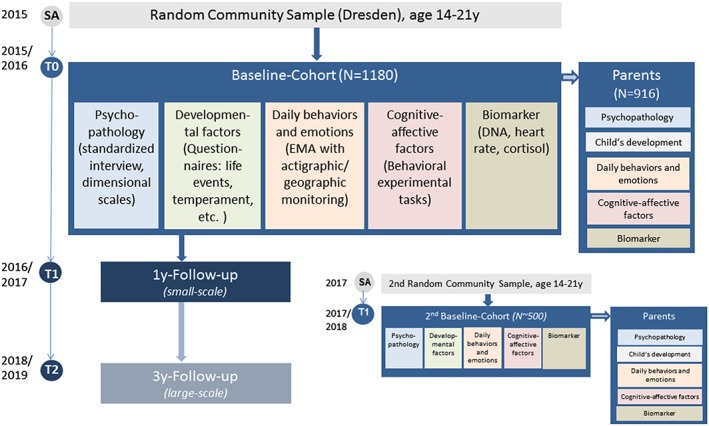
Design of the BeMIND study

The Behavior and Mind Health (BeMIND) study is designed as a cohort study in a general population sample of adolescents and young adults from Dresden, a major city in the eastern part of Germany. The study comprises a baseline investigation and 1‐ and 3‐year follow‐up investigations to examine developmental trajectories of mental disorders and health risk behaviors related to noncommunicable somatic disease (Figure 1). In addition, the study includes a family study component.

To increase the overall sample size and to allow for replications of exploratory findings, a second smaller baseline‐cohort has been independently sampled approximately 2 years after the original baseline‐cohort (not detailed herein). The study protocol and its amendments were approved by the ethics committee of the Technische Universität Dresden (TUD; EK38110214).

### 2.2 Sampling

The 14‐ to 21‐year‐old population living in Dresden, Germany, represents the study's target population. An age‐ and sex‐stratified random sample of 14–21 year olds was drawn from the population registry of the city of Dresden in 2015 with the aim to recruit ~1,000 adolescents and young adults to become part of the prospective‐longitudinal BeMIND study. Because it was deemed more important to ensure a sufficient sample size for each age group than to resemble the age/sex distribution of the target population (considerably more young adults than adolescents live in Dresden due to two large higher education institutions), younger individuals were oversampled. Sample size was determined based on a priori power calculation; Data S1A provides for select core research questions the power based on the final baseline sample size.

Eligibility to participate in the BeMIND study required living in a household in Dresden during the time of the field work and being between ages 14 and 21. Exclusion criteria were institutionalization and insufficient German language skills. Besides the 14–21 year olds, all parents willing and able to participate in the family study component were assessed at baseline with similar procedures. A smaller scale 1‐year and a large‐scale 3‐year follow‐up investigations are conducted in which all baseline participants are approached again.

### 2.3 Field work and procedures

Address lists of randomly selected adolescents and young adults with primary living address in Dresden were provided by the city's resident registry office. In case of minors, names and address of the legal guardians were also provided. A personal invitation letter was sent by the BeMIND study team with information about the study, a response sheet, and a postage‐paid return envelope. In the case of minors, letters were addressed to both subjects and parents. The information covered aims, approach, and comprehensiveness of the BeMIND study program; 50€ were offered for participating in all baseline study components (overall 6–10 hr on two assessment days and during 4 days in real life; details below); parents were offered 30€. Individuals/families indicated their interest to participate and contact information or the reasons for nonparticipation on the response sheet. A maximum of two reminder letters was sent to subjects/families if there had been no response after 3–4 weeks. No initiating personal or telephone contact could be made by study staff after nonresponse due to legal regulations. Nonparticipants were asked to return a brief nonresponder questionnaire.

With subjects who indicated interest to participate, a personal appointment was made in order to provide detailed study information and to obtain written informed consent/assent. In minors, all legal guardians provided written informed consent. Assessments were then conducted at the Center for Clinical Epidemiology and Longitudinal Studies at TUD. When participation at the research facility was not possible or desired by the subject, subjects' own residences were used.

At baseline, subjects participated in a clinical‐diagnostic assessment (Day 1), in a laboratory assessment approximately 1 week later (Day 2), and in an Ecological Momentary Assessment (EMA) during 4 days in real life and an online questionnaire assessment in between these personal appointments. Biological/physiological data were collected during the EMA period (saliva and heart rate) and at the second personal appointment (blood/buccal, hair, anthropometric measures, and blood pressure).

For the supplementary family study, parents of minors were invited simultaneously to the index subjects because the contact information was provided by the resident registry office; parents of 18+ year olds were invited by written invitation letter if contact information was provided by the index participant on site. Assessments and procedures for parents were similar to those of the index subjects. If a full assessment of parents was not possible (e.g., distant residence), parents were invited to complete a web‐based assessment focusing on early developmental factors of the index child.

### 2.4 Assessments and measures

An overview of the BeMIND baseline assessments is provided in Table 1. Measures were chosen to cover constructs of mental health/disorders and health behaviors (outcomes) as well as putative risk and protective factors (exposures) based on literature and utility/feasibility (psychometric properties, coverage, and brevity).

**Table 1:** Overview of BeMIND baseline assessments

Category/construct	Target population	Assessment time	Assessment mode	Coverage (time frame)	Measure
Sociodemographic variables					
Sex	A; P	D1	I	Current	DIA‐X‐5 Section A/family tree chart
Age	A; P	D1	I	Current	DIA‐X‐5 Section A/family tree chart
Education	A; P	D1; O	I; Q	Lifetime/current	DIA‐X‐5 Section A
Financial situation	A; P	D1	I; Q	Current	DIA‐X‐5 Section A
Employment	A; P	D1	I; Q	Current	DIA‐X‐5 Section A
Marital status	A; P	D1	I; Q	Current	DIA‐X‐5 Section A
Living situation	A; P	D1	I; Q	Current	DIA‐X‐5 Section A
Age of parents at index's birth	P	D1; O	Q	Past	Items used in EDSP
Index's familial and financial situation during childhood	A; P	D1; O	Q	Past	Items used in BELLA/EDSP
Index's educational trajectory	P	D1; O	Q	Past	Items used in EDSP
Family composition	A; P	D1	I	Current	Family tree chart
Health and health behavior					
Psychopathology—categorical					
Symptoms, syndromes, and diagnoses of mental disorders (anxiety, depressive, bipolar, substance use, eating, stress‐related, obsessive–compulsive spectrum, psychotic, somatic symptom, attention‐deficit/hyperactivity, intermittent explosive, and disruptive/antisocial behavior disorders)	A; P	D1	I; Q	Lifetime; past 12 months	DIA‐X‐5; self‐administered questionnaire for parents
Index's symptoms, syndromes, and diagnoses of childhood mental disorders (attention deficit/hyperactivity, conduct, oppositional defiant, intermittent explosive, separation anxiety, generalized anxiety, depressive, and disruptive mood dysregulation disorder)	P	D1; O	Q	Lifetime	Items based on DIA‐X‐5
Diagnosed mental disorders	A; P	O	Q	Lifetime; past 12 months	Adapted items from DEGS
Index's diagnosed mental disorders	P	D1; O	Q	Lifetime; past 12 months	Items used in EDSP
Self‐harm behavior	A; P	D1	Q	Lifetime; past 12 months	Adapted items of the self‐injurious thoughts and behaviors interview for self‐harming behavior (SITB) and Functional Assessment of Self‐Mutilation (FASM)
Suicidal behavior	A; P	D1	I; Q	Lifetime; past 12 months	DIA‐X‐5; adapted version of the Questionnaire on Suicidal attempts
Symptoms, syndromes, and diagnoses of relatives' mental disorders (index's parents and their partners, grandparents, and siblings)	A; P	D1	I; Q	Lifetime; past 12 months	Items within DIA‐X‐5; self‐administered questionnaire for adolescents and parents
Psychopathology—dimensional					
Risk for autism spectrum disorders (ASD)	P	D1	Q	Age 2	Short Version of the Modified Checklist for Autism in Toddlers (M‐Chat)
Mental health of parents	P	D1; O	Q	Ages 0 to 5	Items used in EDSP
Alcohol consumption	F‐A	D1	Q	Past 12 months	Alcohol Use Disorders Identification Test (AUDIT‐G‐L)
Premenstrual symptoms	F‐A	D1	Q	Past 12 months	Shortened Version of the Premenstrual Symptom Scale
Anxiety	A	D1	Q	Past 4 weeks	Cross‐cutting Dimensional Severity Measure for Anxiety (Cross‐D)
Agoraphobia	F‐A	D1	Q	Past 4 weeks	DSM‐5 Disorder‐Specific Severity Measure for Agoraphobia (AG‐D)
Generalized anxiety	F‐A	D1	Q	Past 4 weeks	Generalized Anxiety Disorder Dimensional Scale (GAD‐D)
Illness anxiety	F‐A	D1	Q	Past 4 weeks	DSM‐5 Level 2 Cross‐Cutting Symptom Measure—Illness Anxiety Disorder (IA‐D)
Panic	F‐A	D1	Q	Past 4 weeks	DSM‐5 Disorder‐Specific Severity Measure for Panic Disorder (PD‐D)
Separation anxiety	F‐A	D1	Q	Past 4 weeks	DSM‐5 Disorder‐Specific Severity Measure for Separation Anxiety Disorder (SepA‐D)
Social anxiety	F‐A	D1	Q	Past 4 weeks	DSM‐5 Disorder‐Specific Severity Measure for Social Anxiety Disorder (SAD‐D)
Specific phobia	F‐A	D1	Q	Past 4 weeks	DSM‐5 Disorder‐Specific Severity Measure for Specific Phobia (SP‐D)
Depression	A	D1	Q	Past 2 weeks	Patient Health Questionnaire‐9 (PHQ‐9)
Repetitive thoughts and behaviors	F‐A	D1	Q	Past 4 weeks	DSM‐5 Level 2 Cross‐Cutting Severity Measure ‐ Repetitive Thoughts and Behaviors‐Adult (adapted from the Florida Obsessive–Compulsive Inventory (FOCI) Severity Scale [Part B])
Irritability	A	D1	Q	Past week	DSM‐5 Level 2 Cross‐Cutting Symptom Measure ‐ Irritability, Child Age 11–17 (Affective Reactivity Index [ARI])
Mania	A	D2; DL	Q; EMA	Past week; since last beep	DSM‐5 Level 2 Cross‐Cutting Symptom Measure ‐ Mania (Altman Self‐Rating Mania Scale [ASMR, adapted items in EMA])
Mental health problems	A; P	D2	Q	Past week	DSM‐5 Self‐Rated Level 1 Cross‐Cutting Symptom Measure
Index's mental health problems	P	D1; O	Q	Past 6 months	Strength and Difficulties Questionnaire extended (SDQ)
Emotional distress—anger	A; P	D2; DL; DL‐N	Q; EMA	Past week; since last beep; past day	DSM‐5 Level 2 Cross‐Cutting Symptom Measure ‐ Anger (PROMIS Emotional Distress ‐ Anger ‐ short‐form)
Emotional distress—anxiety	A; P	D2; DL; DL‐N	Q; EMA	Past week; since last beep; past day	DSM‐5 Level 2 Cross‐Cutting Symptom measure ‐ Anxiety (PROMIS Emotional Distress ‐ Anxiety ‐ short‐form)
Emotional distress—depression	A; P	D2; DL; DL‐N	Q; EMA	Past week; since last beep; past day	DSM‐5 Level 2 Cross‐Cutting Symptom Measure ‐ Depression (PROMIS Emotional Distress ‐ Depression ‐ short‐form)
Compulsive internet use	F‐A	O	Q	General	Compulsive Internet Use Scale (CIUS)
Video game dependency	F‐A	O	Q	General	Video Game Dependency Scale (KFN‐CSAS‐II)
Physical health					
Natal complications	P	D1; O	O	past	Items used in EDSP
Physical health of parents	P	D1; O	Q	Past (ages 0 to 5)	Items used in EDSP
Somatic symptoms, sensory symptoms, and physical disabilities	P	D1; O	O	Lifetime	Items used in EDSP
Chronic physical diseases (allergies, asthma, cardiovascular diseases, etc.)	A; P	O	Q	Lifetime, past 12 months	Items adapted from KIGGs and DEGS
Chronic disease	A; P	O	Q	Current	Item of the Minimum European Health Module (MEHM)
illness	A; P	D2	BP	Past 3 months	Hair sample protocol
Physical health problems	A; P	DL; DL‐N	EMA	Since last beep; past day	Self‐developed item
Disability	A; P	O	Q	Past month	World Health Organization Disability Assessment Schedule II (WHODAS 2.0)
Impairment days (complete and partial) due to physical and mental health problems	A; P	D1	I; Q	Past 4 weeks	DIA‐X‐5 Section Q
Physical and mental health impairment	A; P	D1	Q	Past 12 months	Adapted Sheehan Disability Scale (SDS)
Sick days	A	O	Q	Past 12 months	Item used in DEGS
Pain intensity	A; P	D2	Q	Past week	PROMIS‐29 Profile v2.0 (pain intensity)
Pain tolerance	A	D2	Q	General	Items of the Capability for Suicide Questionnaire (CSQ)
Pubertal stage	F‐A	O	Q	Current	Tanner Stages Test
Puberty: acne, hair growth, vocal change in boys, age of first menstruation, and regular menstruation	A	O	Q	Lifetime; current	Self‐developed items and item of Pubertal Developmental Scale (PDS) and item used in KIGGS survey
Reproductive health: sexual contact, use of birth control, and pregnancy in girls	A	O	Q	Lifetime; current	Items used in HBSC Survey and in DEGS
Sight or hearing problems	A; P	D1; O	Q	Current	Self‐developed items
Anthropometry					
Height	A; P	D2	AM	Current	Without shoes using a standard stadiometer
Weight	A; P	D2	AM	Current	Without shoes and heavy clothing using a standard digital scale
Hip circumference	A; P	D2	AM	Current	Standard linear measure
Waist circumference	A; P	D2	AM	Current	Between the lower rib margin and the iliac crest in the horizontal plane using a standard linear measure
Head circumference	A	D2	AM	Current	Standard linear measure
General health					
Early developmental stages	P	D1; O	Q	Early childhood	Early Development & Home Background (EDHB) Form
Subjective general health	A; P	O	Q	General	Items of the Short Form Health Survey (SF‐36)
Subjective mental and physical health	A; P	O	Q	General	Self‐developed items
Service utilization and treatment					
Help‐seeking behavior in infancy and childhood	P	D1; O	Q	Past	Item used in EDSP
Mental health care use	A; P	D1	I; Q	Lifetime	DIA‐X/5I Section Q
Treatment for mental health problems with psychotherapy/medication	A; P	D1	I; Q	Lifetime	DIA‐X/5 Section Q
Emergency care visits	A	O	Q	Past 12 months	Item used in DEGS
Health care use: practitioners	A	O	Q	Past 12 months	Adapted items used in DEGS
Preventive service use	A	O	Q	Past 12 months	Adapted items used in DEGS
Medication use	A; P	D2	D2	Past 7 days	Self‐developed item
Lifestyle					
Diet and substance use					
Diet—attitude towards diet	A	D2	Q	General	Items of the Eating Behavior and Weight Problems Inventory (EWI)
Diet—habits and problems	A; P	O	Q	General	Items used in HBSC Survey
Diet—behavior	A	DL	EMA	Since last beep	Self‐developed item
Substance use—any, craving	A; P	DL; DL‐N	EMA	Since last beep; past week	Items based on DIA‐X‐5
Substance use—alcohol	A; P	D1; D2	I/Q; BP	Lifetime; past 3 months	DIA‐X‐5 Section I; hair sample protocol
Substance use—psychoactive medication and illicit drugs	A; P	D1; D2	I/Q	Lifetime	DIA‐X‐5 Section L
Substance use—smoking (tobacco)	A; P	D1; D2	I/Q; BP	Lifetime; current	DIA‐X‐5 Section B; hair sample protocol
Substance use—alcohol, benzodiazepine, and cigarettes	A; P	DL	BP	Past day	Saliva sample protocol
Substance use—medication	A; P	DL	BP	Past 7 days	Saliva sample protocol
Activity					
Leisure time activities	A; P	O	Q	General	Adapted items used in NCS‐A
Physical activity and its intensity	A; P	D2	BP	General	Hair sample protocol
Physical activity	A; P	O	Q	Past 3 months	Items used in DEGS and from the GPAQ
Current activities	A; P	DL; DL‐N	EMA	Since last beep; past day	Self‐developed item
Local position and environment	A	DL	EMA	Since last beep	Self‐developed items
Physical activities	A; P	DL; DL‐N	EMA	Since last beep; past day	Self‐developed item
Movement radius and points of interest	A	DL	EMA	Current (continuous over 4 days)	GPS tracking in the smartphone with sampling frequency between 1 and 0.2 Hz
Sleep					
Sleep problems	A; P	O	Q	Lifetime; past month	Self‐developed questionnaire
Sleep disturbance	A; P	O; DL‐M	Q; EMA	Past month; past night	DSM‐5 Level 2 Cross‐Cutting Symptom Measure ‐ Sleep Disturbance (PROMIS Sleep Disturbance short‐form)
Sleep duration	A; P	O	Q	Past month	Item used in DEGS
Day sleep	A; P	DL‐N	EMA	Past day	Self‐developed item
Sleep quantity	A; P	DL‐M	EMA	Past night	Self‐developed items
Objective physical activity					
Blood pressure (systole, diastole, and pulse)	A; P	D2	Bio	Current	Oscillometric digital blood pressure monitor (705IT, OMRON)
Blood/buccal sample	A; P	D2	Bio	Current	Two 9‐ml blood samples by venipuncture (S‐Monovettes; Sarstedt, Nümbrecht); or—alternatively—buccal swabs (Biozyme, Wien) for sampling buccal mucosa
Hair sample with standard hair protocol	A; P	D2	Bio	Past 3 months	Two to three 3‐cm‐long, 3‐mm‐wide hair samples taken scalp‐near from a posterior vertex position
Saliva samples with time recording	A; P	DL	Bio	Current (two subsequent weekdays)	Taken immediately after awakening, 30 min after the first sampling and 30 min before going to bed (Salivettes: Sarstedt, Nümbrecht, Germany; MEMSCAPS (MEMS 6 TrackCap container): Aardex Ltd., Switzerland)
Heart rate/heart rate variability (HRV)	A	DL	EMA	Current (continuous over 4 days)	HRV in millisecond accuracy (Firstbeat Bodyguard 2)
Objective physical activity	A	DL	EMA	Current (continuous over 4 days)	Three‐axis acceleration sensor system with a sampling frequency of 12.5 Hz (Firstbeat Bodyguard 2)
Individual and environmental factors					
Psychological factors					
Comparison of competencies	A	O	Q	General	Extended scale for comparison of competencies (VK+)
Life satisfaction	A; P	O	Q	General	Cantril's self‐anchoring ladder rating of life
Dispositional optimism	A; P	O	Q	General	Life‐Orientation‐Test (LOT‐R)
Locus of control	A; P	O; DL‐N	Q; EMA	General; past day	Internal‐External‐Locus of Control‐4 (IE‐4)
Sense of coherence	A; P	O	Q	General	Item of the Sense of Coherence Scale (SOC‐L9)
Self‐confidence, happiness, and mastery	A; P	D2	Q	General	Self‐developed items
Self‐esteem	A; P	O	Q	General	Single‐Item Self‐Esteem Scale (SISE)
Self‐esteem	A; P	DL‐N	EMA	Past day	Three items of the Rosenberg Self‐Esteem Scale (RSES)
Daily meaning	A	DL‐N	EMA	Past day	Daily Meaning Scale (DMS)
Quality of life	A; P	DL‐N	EMA	Past day	EUROHIS‐QOL 8‐item
Optimism/pessimism	A	DL	EMA	Current	Skala Optimismus‐Pessimismus‐2 (SOP2)
Social parameters					
Social situation of parents	P	D1; O	Q	Ages 0 to 5	Items used in EDSP
Parental style	A	O	Q	Childhood	Measure of Parental Style (MOPS) and adapted items of the Parental Bonding Instrument used in NCS‐A
Attachment	A; P	O	Q	General	Relationship Questionnaire (RQ)
Burdensomeness	A; P	D2	Q	General	Item of the Interpersonal Needs Questionnaire (INQ)
Loneliness and social exclusion	A; P	D2	Q	General	Self‐developed items
Shyness	A; P	D2	Q	General	Self‐developed item
Stigma against mental illness	A; P	D2	Q	General	Self‐developed item
Social support	A; P	O	Q	General	Short form of the social support questionnaire (F‐SOZU)
Partnership and duration	A	O	Q	Lifetime	Adapted items of KIGGS
Important relationships	A	D1	Q	Past 6 months	Items used in EDSP
Social interactions	A; P	DL; DL‐N	EMA	Since last beep; past night	Self‐developed item
Social media use	A	O	Q	Current	Self‐developed items
Social support	A; P	O; DL‐N	Q; EMA	Past day	Oslo 3 Support Scale extended (OSLO ‐3)
Cognition					
Volitional competencies	A; P	O	Q	General	Short form of the Volitional Components Questionnaire (VCQ‐S‐SF)
Attributional style	A; P	O	Q	General	Self‐developed items
Approach and avoidance behavior	A	O; DL	Q; EMA	General; since last beep	Self‐developed items
Volition	A; P	DL; DL‐N	EMA	Since last beep; past day	Self‐developed items
Advantageous and disadvantageous choices	A; P	D2	ET	Current	Novel variant of an intertemporal choice task (ITC)
Context processing, goal maintenance, and updating	A; P	D2	ET	Current	AX continuous performance task (AX‐CP)
Inhibition	A	D2	ET	Current	Number Stroop task (Stroop)
Inhibition	A	D2	ET	Current	Go–nogo task (Go–nogo)
Reactions to emotional faces	A; P	D2	ET	Current	Emotional face approach‐avoidance task (Face AAT)
Speed of processing, visual search, scanning, flexibility, and executive functions	A; P	D2	Test	Current	Trail‐Making Test—A/B (TMT)
Speed of processing	A; P	D2	Test	Current	Number Connection Test (Zahlenverbindungstest [ZVT])
Working‐memory's number storage capacity	A; P	D2	Test	Current	Digit Span Task—forward/backward (DSP)
Emotion					
Emotion regulation	A	O	Q	General	Emotion‐Regulation Skills Questionnaire (SEK/ERSQ)
Emotional pain tolerance	A; P	D2	Q	General	Self‐developed item
Experiential avoidance	A; P	DL; DL‐N	EMA	Since last beep; past day	Adapted items from another EMA study
Positive/negative mood	A; P	DL; DL‐N	EMA	Since last beep; past day	Self‐developed item
Mood	A	DL	EMA	Current	Six‐item short form of the Multidimensional Mood Questionnaire (MDBF)
Temperament/personality					
Behavioral inhibition	P	D1	Q	Ages 0 to 2	Retrospective Infant Behavioral Inhibition Scale (RIBI)
Behavioral inhibition: social/school and fear/illness	A	O	Q	Ages 5 to 16	Retrospective Self Report of Inhibition (RSRI)
Behavioral inhibition/activation	A; P	O	Q	General	Behavioural Inhibition System/Behavioural Activation System scales (BIS/BAS scales)
Big Five personality	A; P	O	Q	General	Short version of the Big Five Inventory (BFI‐10)
Personality: negative affect, detachment, antagonism, disinhibition, and psychoticism	A; P	D1; D2	Q	General	Personality Inventory for DSM‐5—Brief Form (PID‐5‐BF)
Personality: harm avoidance, reward dependence, and sensation seeking	A; P	D2	Q	General	Short form of the Tridimensional Personality Questionnaire (TPQ‐44)
Willingness to take risks	A; P	O	Q	General	Kurzskala Risikobereitschaft‐1 (R‐1)
Intolerance of uncertainty	A	O	Q	General; past day	Short version of the German Intolerance of Uncertainty Scale (IUS‐D)
Coping/resiliency					
Coping	A	O	Q	General	Extended Brief COPE
Resilience	A; P	O	Q	General	Connor–Davidson Resilience Scale (CD‐RISC‐10)
Resilience	A; P	O	Q	Past 3 months	Resilience Scale for Adolescents (READ); Resilience Scale for Adults (RSA)
Resilience	A; P	DL‐N	EMA	Past day	Brief Resilient Coping Scale (BRCS)
Self‐efficacy	A; P	O; DL‐N	Q; EMA	General; past day	Short Scale for Measuring overall Self‐efficacy Beliefs (ASKU)
Stress					
Discomfort intolerance	A; P	D2	Q	General	Four items of the Discomfort Intolerance Scale (DIS)
Childhood trauma/adversity	A	D1	Q	Until age 18	Child‐Trauma Questionnaire extended (CTQ)
Violence against mother or father	A	D1	Q	Until age 18	Adapted items of the Adverse Childhood Experiences Questionnaire (ACE)
Trauma	A; P	D1	I; Q	Lifetime	DIA‐X‐5 Section N
Separation from parents	A; P	D1	Q	Past	Items used in EDSP
Life events and conditions	A	O	Q	Past 5 years	Munich Event list for each year (MEL)
Assaults, aggressive behavior, bullying as victim and perpetrator in real life and social media	A	D1	Q	Past 12 months	Self‐developed questionnaire with items from DEGS
Burdensome events	A; P	D1	I; Q	Past 12 months	DIA‐X‐5 Section AD
School performance	A	O	Q	Past 12 months	Self‐developed items
Work and life conditions	A; P	O	Q	Past 12 months	Self‐developed items
Stress—chronic	A; P	O	Q	Past 3 months	Trier Inventory of the Assessment of Chronic Stress‐Screening (TICS‐SSCS)
Stress—load	A; P	D2	BP	Current; past 3 m	Hair and cortisol sample protocols
Stress—perceived	A; P	O	Q	Past month	Perceived Stress Scale (PSS‐4)
Stress—perceived	A; P	DL‐N	EMA	Past day	Perceived Stress Scale (PSS‐10)
Stress—expected	A; P	DL‐M	EMA	Past night	Self‐developed items
Daily hassles	A	O	Q	Past 2 weeks	Daily Hassles Scale (DH)
Time urgency	A	O	Q	Past 2 weeks	Self‐developed items
Bullying	A; P	DL‐N	EMA	Past day	Self‐developed item
Mood changing events	A; P	DL	EMA	Since last beep	Self‐developed items
Biomaterials and physiological parameters					
Blood pressure (systole, diastole, and pulse)	A; P	D2	Bio	Current	Oscillometric digital blood pressure monitor (705IT, OMRON)
Blood/buccal sample	A; P	D2	Bio	Current	Two 9‐ml blood samples by venipuncture (S‐Monovettes; Sarstedt, Nümbrecht); or—alternatively—buccal swabs (Biozyme, Wien) for sampling buccal mucosa
Hair sample with standard hair protocol	A; P	D2	Bio	Past 3 months	Two to three 3‐cm‐long, 3‐mm‐wide hair samples taken scalp‐near from a posterior vertex position
Saliva samples with time recording	A; P	DL	Bio	Current (two subsequent weekdays)	Taken immediately after awakening, 30 min after the first sampling and 30 min before going to bed (Salivettes: Sarstedt, Nümbrecht, Germany; MEMSCAPS (MEMS 6 TrackCap container): Aardex Ltd., Switzerland)
Heart rate/HRV	A	DL	EMA	Current (continuous over 4 days)	HRV in millisecond accuracy (Firstbeat Bodyguard 2)
Objective physical activity	A	DL	EMA	Current (continuous over 4 days)	Three‐axis acceleration sensor system with a sampling frequency of 12.5 Hz (Firstbeat Bodyguard 2)

*Note.* Target population: A: adolescents and young adults (index subjects); F‐A: filtered adolescents (i.e., by age, video game use); P: parents. Assessment time: D1: first assessment day; D2: second assessment day; DL: in daily life between D1 and D2; DL‐M: morning assessment in daily life between D1 and D2; DL‐N: night assessment in daily life between D1 and D2; O: Online‐questionnaire between D1 and D2. Assessment mode: Q: questionnaire; I: interview; EMA: ecological momentary assessment; AM: anthropometric measures; BP: biological sample protocol; ET: experimental tasks. BELLA: mental health module (BELLA study) within the German Health Interview and Examination Survey of Children and Adolescents; DEGS: German health interview and examination survey for adults; EDSP: Early Developmental Stages of Psychopathology study; HBSC: Health Behaviour in School‐aged Children study; KIGGS: German Health Interview and Examination Survey of Children and Adolescents; NCS‐A: National Comorbidity Survey Replication Adolescent Supplement. References for the Measures in Table 1 are available from the authors upon request.

#### 2.4.1 Standardized assessment of mental disorders

Diagnostic status of index subjects was determined using an updated version of the Munich Composite International Diagnostic Interview (DIA‐X/M‐CIDI; Wittchen & Pfister, 1997). The DIA‐X/M‐CIDI provides lifetime and 12‐month diagnoses for a wide range of mental disorders including anxiety, depressive, bipolar, substance use, somatic symptom/somatoform, psychotic disorders, and eating disorders and was originally designed to assess DSM‐IV (APA, 2000) and ICD‐10 (WHO, 1993) criteria. The updated version was created to asses diagnoses according to DSM‐5 (APA, 2013) and a broader range of disorders (e.g., also disruptive behavior, attention‐deficit/hyperactivity, and impulse‐control disorders; DIA‐X‐5; Hoyer et al., submitted). The fully standardized computer‐assisted personal interviews were conducted face‐to‐face by trained clinical (psychology/medical) interviewers. Supporting lists and dimensional symptom scales were applied via tablet computers. Adjacent to each diagnostic section, index subject provided family history information on psychopathology of parents, grandparents, siblings, and—if relevant—one significant other person per DIA‐X‐5 stem screening question. The time for the standardized interview at baseline varied broadly depending on the amount of psychopathology reported and the speed in answering tablet‐based questionnaires (1.5–8 hr). For interviews taking 3+ hours, scheduling a separate appointment was offered.

Parents completed a self‐administered short form of the DIA‐X‐5 via tablet questionnaire providing data on own psychopathology. Additional family history information was assessed from participating parents and—whenever possible—also from the index subject using an extended family history module applied via tablet‐questionnaire covering mental disorders of the index child's grandparents (biological‐/step‐/adoptive) parents, siblings, and—if relevant—one other significant person. Parents reported also about early childhood disorders of the index child. Family history items were designed using a modified version of the Family‐History‐Research‐Diagnostic‐Criteria (Lieb, Isensee, von Sydow, & Wittchen, 2000; Merikangas et al., 1998) as a basic model.

#### 2.4.2 Ecological momentary assessment

In the EMA assessment (Shiffman, Stone, & Hufford, 2008), index subjects answered questions presented via a self‐developed study smartphone app on eight occasions per day over the course of four consecutive days (two weekdays and the weekend). Three survey sets were configured (one morning, six midday, and one evening assessment), each containing 203–248 items, most of which related to the time window since the previous assessment. Implemented branching rules allowed an adaptive answering of the questions so that the study load and time burden could be reduced (~3 min per assessment). The assessments covered current mood and emotions, perceived stress, substance use, daily activities, approach/avoidance behaviors, eating behavior, and physical activity (Table 1). Additionally, sleep behavior and quality during the last night were assessed in the morning and quality of life and subjective stress over the course of the day in the evening assessment. Using GPS tracking, the geographic position of the subject was continuously recorded to investigate the movement radius and points of interest. Furthermore, heart rate/heart rate variability (HRV) and objective movement data were continuously recorded via an integrated HRV and acceleration sensor system (Firstbeat Bodyguard 2; Parak & Korhonen, 2015).

Subjects were informed by trained staff about the EMA study procedures including a presentation of handling the smartphone and HRV sensor. In order to optimally tailor the EMA assessments to the subjects' everyday life, sleep times and periods during which the subjects did not want to be disturbed were requested. An individual reminder scheme was created for each subject and transferred to the smartphone. Reminders were distributed symmetrically throughout the day, taking into account the “nondisturb” times to cover the entire day's course. Participants were allowed to postpone each survey prompted by an acoustic signal three times by 5 min or omit the questionnaire if it was not possible to complete.

On the day before the first assessment, three questionnaire sets were presented in order to familiarize participants with the EMA modality. On the day after the last assessment, a postassessment questionnaire asked for specific impressions and difficulties during the assessment period.

Collected data were stored locally on the smartphone and HRV device and transferred to the study server after the participant returned the equipment to the study center. GPS data were recorded on the smartphone (sampling frequency 0.2 Hz), and HRV (in millisecond accuracy) and physical activity data (three‐axis acceleration sensor motion data with a sampling frequency of 12.5 Hz) were recorded continuously over the 4‐day period. Participants were asked to wear the HRV sensor over the entire EMA period (including the night), which was attached to the skin with electrodes on the upper body and only to be taken off before contact with water. Replacement electrodes were made available to the subjects in sufficient quantities.

On the two weekdays of the EMA period, the awakening reaction and the daily variation of cortisol levels was determined by collecting saliva samples immediately after awakening, 30 min later, and 30 min before bedtime. Subjects were shown how to provide the saliva samples using Salivettes (Sarstedt, Nümbrecht, Germany) and received reminders by the smartphone. The actual saliva sampling time was recorded with the use of MEMSCAPS (MEMS 6 TrackCap container, Aardex Ltd., Switzerland). Participants were asked to store the saliva samples immediately in their freezer before returning them at the second personal appointment to the study personnel, who immediately stored the samples in a laboratory freezer. After thawing, saliva samples were centrifuged for 10 min at 4,000 rpm and cortisol concentrations were determined using a commercially available chemiluminescence assay (CLIA, IBL‐Hamburg, Germany).

Participating parents completed a paper‐and‐pencil‐based EMA twice a day (morning and evening) over the course of 4 days (two weekdays and weekend) and also provided three saliva samples on the two weekdays.

#### 2.4.3 Biosamples and anthropometric measurements

Besides saliva samples over the course of two EMA days for diurnal cortisol analyses, a hair sample was taken by trained study personnel for assessment of long‐term cortisol secretion both from index subjects and participating parents (Kirschbaum, Tietze, Skoluda, & Dettenborn, 2009). Two to three 3‐cm‐long hair samples were collected, scalp‐near from a posterior vertex position. The selected hair was about the diameter of a pencil (~3 mm) and stored in foil. After the hair sample, participants filled in questions about hair treatments (e.g., dyeing), alcohol consumption, smoking, sporting activities, and recent illnesses or stressful situations (Stalder & Kirschbaum, 2012).

In addition, two 9‐ml EDTA blood samples were collected by venipuncture from index subjects and participating parents using the vacuum method for DNA analyses (S‐Monovettes; Sarstedt, Nümbrecht) and were stored at −80°C. For participants not concurring with the blood withdrawal, buccal swabs (Biozyme, Wien) for sampling buccal mucosa were offered.

Standard digital scales were used with index‐subjects and participating parents for anthropometric measures (weight, height, and waist circumference). Systolic and diastolic blood pressure were measured after a resting period of at least 5 min, and three times on the arm without blood sampling of seated subjects using an oscillometric digital blood pressure monitor (705IT, OMRON).

#### 2.4.4 Questionnaire assessments

Index subjects completed a range of questionnaires during the personal assessments in the study center and during a web‐based online assessment between the two personal appointments. Questionnaires assessed putative distal and proximal individual and environmental risk and protective factors covering constructs from various domains (see Table 1). The total time for filling in the questionnaires was approximately 60–90 min online and 30–60 min on site.

Participating parents completed a shorter version of the questionnaire assessment and provided additional information on pregnancy, birth, and early development of the index child.

#### 2.4.5 Behavioral tests and tasks

Index subjects completed paper‐and‐pencil tests and computerized tasks on executive functioning, cognitive control, and decision making during the second personal appointment. The total time for this behavioral experimental assessment was ~70 min.

Three *paper‐and‐pencil tests* were conducted by trained examiners. For a nonlanguage‐based measure of intelligence (“speed of processing”), the number connection test (German ZVT; Oswald & Roth, 1987) was conducted. The ZVT is comparable with the trail‐making test (Reitan, 1955), which was also applied. To measure working‐memory's number storage capacity, a digit span task was used (Richardson, 2007).

The matlab‐programmed *task battery* included five tasks that were presented in the same order to every subject and that started automatically. Subjects were introduced to the set up by trained study personnel. During task battery performance, subjects were alone in a quiet room and allowed to do self‐paced breaks between the individual tasks. The tasks included the number Stroop task (Stroop, 1932) to measure inhibition (using a mouse‐click version in contrast to common keyboard‐based response), the emotional face approach‐avoidance task (Face‐AAT; Heuer, Rinck, & Becker, 2007) to investigate reactions to emotional faces, the AX continuous performance task (AX‐CP; Cohen, Barch, Carter, & Servan‐Schreiber, 1999) to measure context processing, goal maintenance and updating, a novel variant of an intertemporal choice task (ITC, Scherbaum, Dshemuchadse, Leiberg, & Goschke, 2013) to measure individually determined advantageous and disadvantageous choices, and a go–nogo task (Wolff et al., 2016) to measure inhibition.

Participating parents completed the same paper‐and‐pencil tests and a shortened task battery (Face‐AAT, AX‐CP, and ITC).

### 2.5 Quality assurance

To ensure high data quality different steps were followed including (a) handing the operation manual with standard operating procedures for recruitment, assessments, and data management to every new staff member; (b) intensive recruitment, interviewer, and other assessment training and certification for new staff including supervision by scientific project members as long as required to ensure high data quality; (c) refreshment trainings for staff every 4–6 months; (d) systematic monitoring of assessments with spot checks throughout the field phase followed by individual feedback to staff members and—if required—additional training; (e) supervisions with the study PI regarding the clinical interviews; and (f) systematic checks of data regarding completeness, consistency, and plausibility.

Prior to the study, all assessments and study procedures were tested for feasibility, practicability, and time requirements. Novel assessments were also checked for reliability and validity. The entire study procedure was piloted with 20 subjects from the general population. To avoid false or missing values and for a time‐economic study, execution value ranges were defined and branching rules were applied. A comprehensive IT infrastructure has been developed for several study aspects such as recruitment, participant management, scheduling appointments, data entry, and integration.

## 3 RESULTS

### 3.1 Recruitment flow and study population

**Figure 2:**
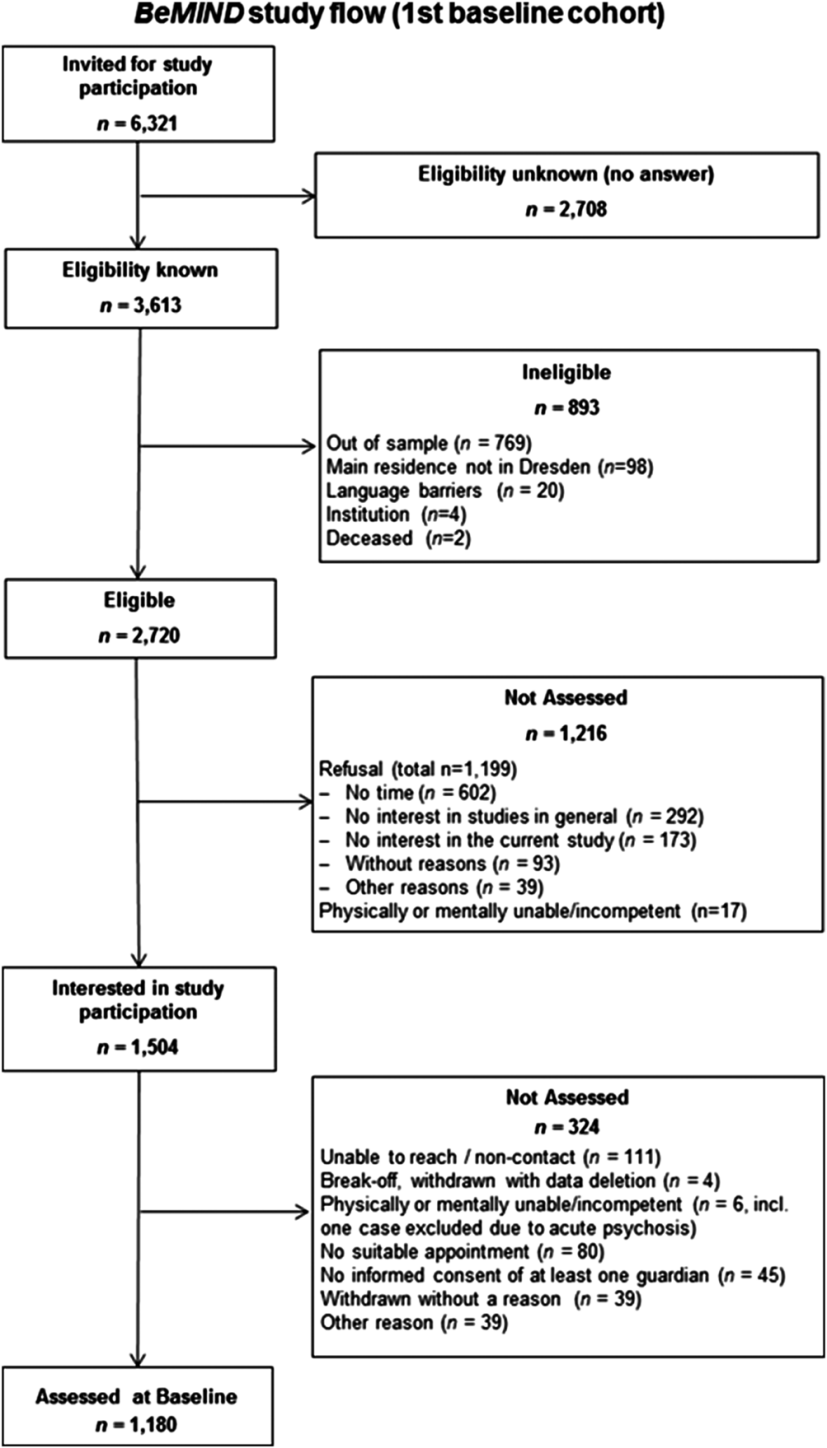
Flow of participant recruitment in the BeMIND study

Invitation letters were subsequently sent to overall 6,321 sampled individuals/families. Of these, 14.1% were ineligible, mostly due to the fact that they were not residing under the provided address (Figure 2). Of the remaining 5,428 individuals, 1,180 were assessed resulting in a minimum response (participation) proportion of 21.7% (AAPOR, 2016: formula RR1). The cooperation rate among those with known eligibility was 43.4% (formula COOP1). The main reason for nonparticipation was refusal, mostly due to lack of time or interest, followed by failure to contact and arranging suitable appointments; 42.8% of all invited individuals/families did not answer the invitation letter, two reminder letters, and an anonymous nonresponder questionnaire. Assuming that the proportion of eligible subjects in these cases with unknown eligibility is the same as the proportion of eligible subjects among those with known eligibility, the estimated overall response proportion was 24.8% (formula RR3) and the overall cooperation rate was 49.5% (formula COOP3).

Table 2 shows the sample and the total population of 14‐ to 21‐year‐old people in Dresden by age and sex. Participation was generally higher in females than males.

**Table 2:** Demographic distribution of the adolescent/young adult population in the city of Dresden, the potentially eligible sample, and the participants unweighted and weighted

	Population in Dresden (date 2015‐12‐31)		Potentially eligible sample (age at date of postal invitation)		Final study sample (assessed participants)				
	*n*	%	*n*	%	*n*	%	*n* weighted	% weighted	Minimum response proportion
Total^a^	37,359	100.0	5,428	100.0	1,180	100.0	1,180.0	100.0	21.7
14	3,878	10.4	782	14.4	146	12.4	122.5	10.4	18.7
15	3,971	10.6	787	14.5	196	16.6	125.4	10.6	24.9
16	3,804	10.2	769	14.1	167	14.2	120.2	10.2	21.7
17	3,674	9.8	759	14.0	126	10.7	116.0	9.8	16.6
18	4,437	11.9	602	11.1	160	13.6	140.1	11.9	26.6
19	5,431	14.5	583	10.7	128	10.8	171.5	14.5	22.0
20	5,879	15.7	580	10.7	134	11.4	185.7	15.7	23.1
21	6,285	16.8	566	10.5	123	10.4	198.5	16.8	21.7
Males	19,323	100.0	2,725	100.0	495	100.0	610.3	100.0	18.2
14	2,018	10.4	386	14.1	67	13.5	51.7	10.4	17.4
15	2,088	10.8	417	15.3	90	18.2	53.5	10.8	21.6
16	1,998	10.3	380	13.9	71	14.3	51.2	10.3	18.7
17	1,878	9.7	383	14.1	51	10.3	48.1	9.7	13.3
18	2,280	11.8	307	11.3	68	13.7	58.4	11.8	22.1
19	2,818	14.6	283	10.4	41	8.3	72.2	14.6	14.5
20	2,999	15.5	295	10.8	55	11.1	76.8	15.5	18.6
21	3,244	16.8	274	10.1	52	10.5	83.1	16.8	19.0
Females^a^	18,036	100.0	2,703	100.0	685	100.0	569.7	100.0	25.3
14	1,860	10.3	396	14.7	79	11.5	70.6	10.3	19.9
15	1,883	10.4	370	13.7	106	15.5	71.5	10.4	28.6
16	1,806	10.0	389	14.3	96	14.0	68.6	10.0	24.7
17	1,796	10.0	376	13.9	75	10.9	68.2	10.0	19.9
18	2,157	12.0	295	11.0	92	13.4	81.9	12.0	31.2
19	2,613	14.5	300	11.1	87	12.7	99.2	14.5	29.0
20	2,880	16.0	285	10.6	79	11.5	109.4	16.0	27.7
21	3,041	16.9	292	10.8	71	10.4	115.5	16.9	24.3

a One participant was at age 22 at the time of interview but is counted at age 21 in all analyses.

In order to improve representativeness of the sample, we apply sample weights. The sample is divided into 16 strata according to the 16 possible combinations of sex and age. The sample weights are calculated so that after weighting adjustment, the relative sample frequencies of these groups equal the corresponding relative frequencies of the strata in the population of the 14‐ to 21‐year‐old people of Dresden (the target population). Note that this accounts for (a) intended (sampling probabilities differing over age groups, by design) and (b) unintended discrepancies. The distribution of other determinants of participation is also adjusted for to an extent that these are associated with sex and age. The age and sex distribution of the target population were taken from the Registration Office of the city of Dresden (Landeshauptstadt Dresden‐Kommunale Statistikstelle, 2016b) and can be considered highly accurate.

### 3.2 Sample characteristics of index subjects

The mean age of the *N* = 1,180 index participants at baseline was 17.3 years (*SD* 2.3) and was similar for boys (17.1, *SD* 2.3) and girls (17.4, *SD* 2.2). As shown in Table 3, most participants had German Nationality (97.5%); 61.2% still went to school, 19.6% were university students; 99.3% of the index subjects had never been married, and 74.7% lived with a parent.

**Table 3:** Demographic sample characteristics of BeMIND index subjects at baseline (*N* = 1,180)

	Total			Age 14–17			Age 18–21			Males			Females		
	*N*	%	%w	*N*	%	%w	*N*	%	%w	*N*	%	%w	*N*	%	%w
Age															
14–17 years	635	53.8	41.0	635	100.0	100.0	0	0.0	0.0	279	56.4	41.3	356	52.0	40.7
18–21 years	545	46.2	59.0	0.0	0.0	0.0	545	100.0	100.0	216	43.6	58.7	329	48.0	59.3
Sex															
Male	495	41.9	51.7	279	43.9	52.1	216	39.6	51.5	495	100.0	100.0	0	0.0	0.0
Female	685	58.1	48.3	356	56.1	47.9	329	60.4	48.5	0	0.0	0.0	685	100.0	100.0
German Nationality															
Yes	1,150	97.5	97.1	621	97.8	97.6	529	97.1	96.8	478	96.6	96.3	672	98.1	98.1
No	30	2.5	2.9	14	2.2	2.4	16	2.9	3.2	17	3.4	3.7	13	1.9	1.9
Living arrangement															
With parents	881	74.7	65.1	623	98.1	98.0	258	47.3	42.1	383	77.4	66.7	498	72.7	63.3
Alone	109	9.2	12.5	5	0.8	0.8	104	19.1	20.6	36	7.3	10.6	73	10.7	14.5
With partner	50	4.2	5.4	2	0.3	0.3	48	8.8	8.9	10	2.0	3.0	40	5.8	7.9
Other	140	11.9	17.1	5	0.8	0.9	135	24.8	28.4	66	13.3	19.7	74	10.8	14.4
Education															
Low	25	2.1	2.3	12	1.9	1.9	13	2.4	2.6	15	3.0	3.2	10	1.5	1.3
Middle	233	19.7	18.6	158	24.9	24.7	75	13.8	14.3	101	20.4	18.7	132	19.3	18.4
High	881	74.7	76.4	433	68.2	68.4	448	82.2	82.0	362	73.1	75.7	519	75.8	77.1
Other	41	3.5	2.8	32	5.0	5.0	9	1.7	1.2	17	3.4	2.3	24	3.5	3.2
Employment															
School	722	61.2	48.7	604	95.1	94.6	118	21.7	16.8	316	63.8	49.0	406	59.3	48.4
University	231	19.6	28.6	0	0.0	0.0	231	42.4	48.6	104	21.0	31.6	127	18.5	25.5
Job training	99	8.4	10.0	16	2.5	2.9	83	15.2	15.0	33	6.7	8.8	66	9.6	11.4
Employed	61	5.2	6.9	3	0.5	0.7	58	10.6	11.2	24	4.8	6.5	37	5.4	7.3
Unemployed	19	1.6	1.6	5	0.8	0.8	14	2.6	2.2	4	0.8	0.8	15	2.2	2.4
Other	48	4.1	4.1	7	1.1	1.0	41	7.5	6.2	14	2.8	3.3	34	5.0	4.9
Social class															
Lowest	23	2.0	2.6	6	1.0	1.1	17	3.1	3.6	10	2.1	2.8	13	1.9	2.4
Lower middle	144	12.5	14.6	48	7.8	7.9	96	17.7	19.1	60	12.4	14.8	84	12.5	14.4
Middle	710	61.5	60.6	391	63.7	63.8	319	59.0	58.4	300	62.0	60.8	410	61.1	60.3
Upper middle	272	23.5	21.7	167	27.2	26.9	105	19.4	18.2	112	23.1	21.2	160	23.8	22.4
Upper	6	0.5	0.5	2	0.3	0.4	4	0.7	0.6	2	0.4	0.5	4	0.6	0.6
No information^a^	25	2.1	—	21	3.3	—	4	0.7	—	11	2.2	—	14	2.0	—
Financial situation															
Very bad, bad	80	6.9	8.1	26	4.2	4.1	54	10.0	10.9	39	8.0	9.3	41	6.1	6.9
Neither good nor bad	406	34.9	33.6	225	36.2	35.7	181	33.5	32.1	155	31.9	30.3	251	37.1	37.1
Good	532	45.8	45.8	298	47.9	48.4	234	43.3	44.0	232	47.7	47.8	300	44.4	43.7
Very good	144	12.4	12.4	73	11.7	11.8	71	13.1	12.9	60	12.3	12.6	84	12.4	12.3
No information^a^	18	1.5	—	13	2.0	—	5	0.9	—	9	1.8	—	9	1.3	—
Marital status															
Married	7	0.6	0.6	2	0.3	0.3	5	0.9	0.9	2	0.4	0.5	5	0.7	0.8
Separated/divorced/widowed	1	0.1	0.2	0	0.0	0.0	1	0.2	0.3	1	0.2	0.4	0	0.0	0.0
Never married	1,172	99.3	99.2	633	99.7	99.7	539	98.9	98.8	492	99.4	99.2	680	99.3	99.2

*Note.* *N*: unweighted number; %w: weighted percentage.

a Percentages of main categories are percentages among those with available information and add up to 100% except for rounding residuals, percentage of category “no information” are unweighted percentages among the complete *N* = 1,180 sample.

Table 4 shows demographic characteristics of participants and nonparticipants with returned nonresponder questionnaire and the 14–21 year olds living in Dresden as indicated by the German Microcensus 2014. The Microcensus subsample of 14‐ to 21‐year‐old participants from Dresden (*N* = 345) cannot be as representative for the target population as the whole Microcensus sample is representative for German households, but it is the most reliable source about key sociodemographic characteristics of this age group in Dresden. Table 4 reveals that the proportion of people with high education level is higher in the BeMIND cohort than in the Microcensus‐sample for the 14‐ to 21‐year‐old Dresden population. Additional weighting procedures accounting for the overrepresentation of high education in the BeMIND sample will, therefore, be used for sensitivity analyses in the future. Furthermore, the proportion of people living with parents and the proportion of people attaining school is higher in the BeMIND cohort than in the Microcensus sample. To a certain extent, this just reflects the fact that at age 16–18, people attaining higher secondary education at school (assigned to the high education group) were more likely to participate in the BeMIND study than people already attaining job training (usually assigned to middle education level).

**Table 4:** Characteristics of BeMIND participants in comparison to nonparticipants and the German Microcensus subsample of 14‐ to 21‐year‐old people in Dresden^a^

	Participants (*n* = 1,180)			Nonparticipants with returned short questionnaire (*n* = 664)			Microcensus 2014 (sample of 14‐ to 21‐year‐old people living in Dresden [*n* = 345])	
	*n*	%	%w	*n*	%	%w	*n*	%
Age								
14–17 years	635	53.8	41.0	423	64.5	40.9	128	37.1
18–21 years	545	46.2	59.0	233	35.5	59.1	217	62.9
Unknown^b^	—	—	—	8	1.2	—	—	—
Sex								
Male	495	41.9	51.7	322	49.2	51.7	162	47.0
Female	685	58.1	48.3	333	50.8	48.3	183	53.0
Unknown^b^	—	—	—	9	1.4	—	—	—
Living arrangement								
With parents	881	74.7	65.1				179	51.9
With partner	50	4.2	5.4		Not assessed		19	5.5
Alone/other	249	21.1	29.6				147	42.6
Living with both (biological) parents (only for <18 years)								
No	221	34.8	34.0	152	35.9	—	46	35.9
Yes	414	65.2	66.0	271	64.1	—	82	64.1
Having partner								
No	679	67.3	64.5	480	72.3	70.0		
Yes	330	32.7	35.5	123	18.5	21.5	Not assessed	
Others	0	0.0	0.0	15	2.3	3.5		
Unknown^b^	171	14.5	—	46	6.9	5.0		
Education								
Low	25	2.1	2.3				27	7.8
Middle	233	19.7	18.6		Not assessed		96	27.8
High	881	74.7	76.4				205	59.4
Other	41	3.5	2.8				17	4.9
Employment								
School/University	952	80.8	77.3	538	81.3	76.9	224	65.0
School	722	61.2	48.7	c			132	38.3
University	231	19.6	28.6	c			92	26.7
Job training	99	8.4	10.0	82	12.4	15.7	76	22.0
Employed	61	5.2	6.9	11	1.7	2.3	26	7.5
Unemployed/Other	19	5.7	5.7	22	3.4	5.1	19	5.5
Unknown	—	—	—	11	1.7	—	—	—
Subjective financial situation								
Very bad, bad	80	6.9	8.1	67	11.0	12.6		
Neither good nor bad	406	34.9	33.6	252	41.4	41.8		
Good	532	45.8	45.8	205	33.7	33.6	Not assessed	
Very good	144	12.4	12.4	85	14.0	12.1		
Unknown^b^	18	1.5	—	55	8.3	—		

*Note.* %w: weighted percentage.

a RDC of the Federal Statistical Office and Statistical Offices of the Federal States, Microcensus 2014, survey year 2014, own calculations. The German Microcensus provides official representative statistics of the population in Germany (covering almost completely 1% of German households).

b Percentages of main categories are percentages from those with available information and add up to 100% except for rounding residuals, percentage of category “unknown” is raw percentage from the complete sample.

c School and University are one category in nonresponder questionnaire.

Table 5 shows general health indicators in the BeMIND participants and the nonparticipants with returned nonresponder questionnaire. Participants and nonparticipants are comparable indicating that over 80% show no or only mild interference due to somatic or mental/psychosomatic/substance use problems in the past 12 months. The BMI distribution was also similar. Yet, in the BeMIND cohort, a somewhat larger proportion indicated having had treatment for chronic somatic disease (22.6% vs. 17.4%) or mental health problems (20.7% vs. 11.9%) and reported to be a current smoker (20.7% vs. 11.9%).

**Table 5:** General health characteristics of the BeMIND participants vs. nonparticipants

	Participants (*n* = 1,180)			Nonparticipants with returned short questionnaire (*n* = 664)		
	*n*	%	%w	*n*	%	%w
Past 12‐month interference due to somatic health problems						
None	522	45.8	46.1	348	53.3	49.7
Mild	510	44.7	44.4	211	32.3	35.4
Moderate	96	8.4	8.4	65	10.0	10.0
Severe	12	1.1	1.2	25	3.8	4.3
Very severe	0	0.0	0.0	4	0.6	0.7
Unknown^a^	40	3.4	—	11	1.7	—
Past 12‐month interference due to mental, psychosomatic, or substance use problems						
None	609	53.3	54.6	446	68.2	63.9
Mild	414	36.2	35.4	124	19.0	21.0
Moderate	113	9.9	9.4	60	9.2	11.2
Severe	7	0.6	0.6	21	3.2	3.5
Very severe	0	0.0	0.0	3	0.5	0.4
Unknown^a^	37	3.1	—	10	1.5	—
Ever in treatment due to chronic somatic disease						
No	826	79.0	77.4	505	81.7	82.6
Yes	220	21.0	22.6	113	18.3	17.4
Unknown^a^	134	11.4	—	46	6.9	—
Ever in treatment due to mental, psychosomatic, or substance use problem						
No	924	79.4	79.3	544	87.7	88.1
Yes	240	20.6	20.7	76	12.3	11.9
Unknown^a^	16	1.4	—	44	6.6	—
Current smoker						
No	966	81.9	79.3	566	89.8	87.2
Yes	214	18.1	20.7	64	10.2	12.8
Unknown^a^	0	0.0	—	34	5.1	—
Body mass index (BMI)						
Underweight (BMI < 18.5)	176	15.4	13.4	117	19.0	16.3
Normal weight (18.5 < BMI < 25.0)	843	73.8	74.4	446	72.5	74.3
Overweight (BMI > 25.0)	123	10.8	12.2	52	8.5	9.4
Unknown^a^	38	3.2	—	49	7.4	—

*Note.* %w: weighted percentage.

a Percentages of main categories are percentages from those with available information, percentage of category “unknown” is raw percentage from the complete sample.

### 3.3 Family study

Overall, 709 parents of 549 index participants were directly assessed with similar procedures. Further, 207 parents completed at least a minor assessment via online questionnaire. Thus, 916 parents of 677 index subjects provided data.

### 3.4 Completion of baseline study assessments

Data S1B provides an overview of the numbers of subjects who provided data to the individual baseline study components. Participation in each study component was high with around 90% or more of the subjects providing some or even complete data.

## 4 DISCUSSION

The BeMIND study offers unique features to expand our knowledge on the complex developmental factors of mental disorders and health risk behaviors contributing to somatic diseases. The particular strength of the study is the assessment of psychological and behavioral factors both in real life (via EMA with combined actigraphic/geographic monitoring) and in the laboratory (via experimental tasks)—in addition to the more traditional assessments used in epidemiological research including clinical‐diagnostic and questionnaire assessments relying on subjective retrospective self‐report. Select biologic measures (DNA and stress markers) allow testing for critical interactions with behavioral, psychological, and/or environmental risk/protective factors.

For analysis and interpretation of BeMIND study data, several limitations need to be considered. First of all, the response rate is relatively low (AAPOR‐RR1: 21.7%; RR3: 24.8%). The cooperation rate was more favorable, yet still below 50%. Decreasing participation is a general problem in epidemiologic study and survey research (Galea & Tracy, 2007), a trend that has been continuing in more recent health studies in Germany and other European countries (Loeffler et al., 2015; Scheidt‐Nave et al., 2012; Scholtens et al., 2015; Volkert et al., 2017) with particularly low participation of adolescents and young adults (Keeble, Baxter, Barber, & Law, 2016; Lange et al., 2017). Several aspects may have prevented a more favorable participation rate in our study. First, in the invitation letter, participants were informed that no individual feedback on test results would be possible. Thus, the only incentive was the relatively low financial compensation. Second, due to legal regulations, standard epidemiological procedures such as home visits or telephone contacts for recruitment could not be carried out; the maximum allowed number of written contact attempts was three. Third, because of the prospective‐longitudinal design, we emphasized to recruit individuals willing to become part of a cohort with follow‐up investigations at irregular intervals. Subjects who might have participated in a cross‐sectional but not longitudinal study might thus have refrained from enrollment. Fourth, the study procedures were comprehensive and time‐consuming. To maximize chances for complete data, this information was given upfront in the invitation letter, which might have decreased participation. No time and no interest were the mostly provided reasons for nonparticipation.

A low response rate may not necessarily impair study validity (Morton, Bandara, Robinson, & Carr, 2012). The risk of bias needs to be carefully assessed for each individual research question under analysis. Representativeness may be limited by many factors that could have been related to participation beyond age and sex. In the BeMIND sample, subjects with a higher education appear overrepresented (76.4%w) when compared with Microcensus data (59.4%), which, however, are based on a much smaller sample size (actually below one third of the BeMIND sample size). Therefore, we decided to weight by default for age and sex as a consequence of our sampling scheme only and not additionally for education. However, we will use such a related weighting variable for sensitivity analysis. No comprehensive epidemiologic study can ever achieve complete representativeness, that is, a sample that represents the source or even target population in every aspect. However, bias due to selection strongly depends on the target parameter to be estimated (e.g., prevalence/incidence rate, association, and causal effect). This bias occurs only if determinants of participation are related to the target parameter (e.g., if education is a moderator of the association between an exposure such as a life event and an outcome such as depression). Then the amount of this bias depends on the sign and magnitude of that association and the difference of the determinant's distribution between sample and target population. Roughly speaking, larger bias is more likely when estimating marginal parameters (prevalences/incidences) than when estimating associations or effects (Little, Lewitzky, Heeringa, Lepkowski, & Kessler, 1997); and bias might be further smaller when moderators of associations are investigated. This assumes that the individual variation in a parameter (heterogeneity) decreases with an increasing number of variables involved. In the BeMIND study, these determinants could include concern and experience of mental health issues (with exposed individuals expected to have higher participation rates).

Missing data within the sample is another limitation and source of potential bias. Among BeMIND study participants, completion of individual assessments was generally high. We will check types of missing data (at random/not at random) and apply appropriate methods (e.g., imputation techniques, sensitivity analyses; Pedersen et al., 2017).

Another limitation refers to generalization of the BeMIND results to adolescents and young adults in Germany (or other countries). Given the regionally restricted sample (Dresden, Germany), it is important to consider how Dresden compares with other German regions. The city of Dresden is the capital of one of the 16 states of Germany, located in the east of Germany. Dresden has a total population of 548,800 inhabitants (in 2015 when sampling occurred). In contrast to other, mostly rural areas in the eastern part of Germany, the mean age of the population is relatively low (mean: 42.9 years) and as such rather comparable with other large cities in eastern Germany (such as Berlin) and to most regions in western Germany. Similar applies to population density. Compared with other large German cities, there is a relatively low proportion of migrants (6.2%, Landeshauptstadt Dresden‐Kommunale Statistikstelle, 2016b). The unemployment rate is in the medium range (7.4%, Landeshauptstadt Dresden‐Kommunale Statistikstelle, 2016a). In eastern Germany, including the city of Dresden, there is relatively high population movement, particularly among young adults. For the large‐scale follow‐up assessment, resources for travel are therefore budgeted. The address information is kept up to date by reminding subjects during the regular newsletters to return their new living information to the study center. New addresses will also be obtained from other information sources as available contact persons and—if needed—from the population registry.

To contrast the limitations of the BeMIND study with the strengths, the EMA of mood, emotions, and behaviors in real life with combined objective measures of activity and stress, as well as the laboratory‐based behavioral indicators of cognitive‐affective functioning and decision making go beyond traditional assessments of epidemiological surveys on mental and behavioral health. They allow for novel insights into the dynamic networks of symptoms and behaviors (Borsboom, 2017; Bringmann et al., 2016) as well as their predictors and predictive potential. Thus, the BeMIND study will advance our knowledge on the behavioral and psychological factors contributing to health and disease as adolescents grow into adulthood and provide new avenues for early detection and personalized interventions.

## Supporting information

Data S1. Supporting Information
