# Supplementary material for: The Behavior and Mind Health (BeMIND) study: Methods, design and baseline sample characteristics of a cohort study among adolescents and young adults
Source: Int J Methods Psychiatr Res. 2019 Dec 5;29(1):e1804. doi: 10.1002/mpr.1804 (PMC7051848; doi:10.1002/mpr.1804)
Supplement: Supplementary file 1 — Data S1. Supporting Information [file MPR-29-e1804-s001.docx]

**Supplement A: Power Calculation**

*Prevalence Estimation.* We made use of a simulation of prevalence estimates in the baseline sample of N=1,180 subjects to investigate the reliability of general prevalence estimates of different magnitudes. Pre-specified proportions of the sample were drawn at random to be assigned “diseased”. The pre-specified proportions were chosen in order to yield typical prevalence estimate values as shown in Table A1 after applying the sample weighting scheme described in the paper. We used the svy: tabulate command of Stata 15 (StataCorp., 2017) for calculation of standard errors and 95% confidence intervals. The standard error calculation is based on Taylor-linearized variance estimation.

Table A1. Prevalence estimation

| prevalence | standard error | 95% confidence interval |
| --- | --- | --- |
| 1% | 0.35 | 0.54 2.02 |
| 2.5% | 0.50 | 1.66 3.68 |
| 5% | 0.69 | 3.79 6.54 |
| 10% | 0.98 | 8.27 12.13 |
| 15% | 1.14 | 12.92 17.40 |
| 25% | 1.36 | 22.43 27.76 |

*Sub-group comparison of metric variables.* A typical subgroup comparison of interest could be the comparison of smokers and non-smokers (or certain lifetime disorder vs. no lifetime disorder) within a certain age group. Comparing 16-17-year old smokers (N=54 subjects in baseline sample) with 16-17-year-old non-smokers (N=239 subjects) while assuming a significance level of 5% and a medium effect size for the group difference (Cohen’s d = 0.5) yields a power of 91%. This was calculated using the sampsi command of Stata 15 and without application of sample weighting. In practice we will apply sample weighting and use Taylor-linearized variance estimation or bootstrapping on occasion to estimate confidence intervals of group differences. The power for the significance test will usually be a bit lower after sample weighting is applied. Hence, we assume as a rule of thumb that only group differences of at least medium effect size can be detected in age groups spanning two years of age with sufficient statistical power.

*Interaction between factors.* We consider the complete BeMIND baseline sample (N=1,180) and a significance level of 5%. Suppose we investigate in a diathesis-stress (dual risk) framework the interaction of exposure to divorce or separation of biological parents (early separation, 30%) and the exposure to an additional stressful life event (SLE, 30%) in predicting any anxiety disorder (15% in the reference group without divorce or separation of parents and no SLE). Assuming an odds ratio between early separation and SLE of 1.2; an odds ratio between early separation and any anxiety disorder of 1.5; an odds ratio between SLE and any anxiety disorder of 1.5, and an odds ratio of 2.5 for the interaction of early separation and SLE in predicting any anxiety disorder, this yields a power of 83%. The calculation is based on the method of Demidenko (2008) and assumes no sample weighting procedure. The actual power when we take sample weighting into account will be slightly lower. Similar power is expected for dichotomous predictor and outcome variables in differential susceptibility models (Ellis et al., 2011; Pluess et al., 2015).

*EMA Analysis.* Power calculation for Ecologic Momentary Assessment (EMA) analysis is very difficult due to the complex multilevel structure of the data and because this research field is very new. We would need to base power analysis on extensive data simulations and many hardly justifiable assumptions would need to be specified. Therefore, we simply stress that the BeMIND sample size far exceeds typical sample sizes of available EMA studies in the field (see reviews: Heron et al., 2010, Baltasar-Tello et al., 2018), where the maximum sample sizes of included studies were N=303 and N=214, respectively. A meta-analysis regarding compliance in EMA studies (Jones e al., 2019) reported a mean number of participants of 154.21 (range 10-1054) among 126 studies considered. The BeMIND sample will be one of the highest powered EMA samples available to date for many types of analyses. Two caveats apply: Some other EMA studies have a larger number of assessments per person over a longer timeframe which is advantageous for certain analyses. Moreover, if the subject of interest is a certain type of disorder rather than the general population, then other EMA samples may be more suitable. However, these caveats do not diminish the exceedingly strong statistical foundation provided by the large sample size of the BeMIND sample even for analyses of subgroups. The main focus in future analyses must be given to reducing bias in statistical estimates, because only variance but not bias is reduced by a large sample size.

*Prospective Analysis:* For prospective analyses, power calculations are based on a conservative follow-up sample size of 700. Within this sample, a comparison of incidences of a disorder between baseline lifetime regular smokers vs. baseline lifetime never-regular smokers (91 vs. 609 estimated) yields a power of above 80% if we assume an Odds Ratio of at least 2.0 between the disorder incidence and lifetime regular smoking at baseline. Another typical prospective analysis would be the comparison of change of interval-scaled variables over time. For example, we could ask whether baseline lifetime regular smoking vs. baseline lifetime never-regular smoking predicts change in the performance of the GoNogo Task (measured by balanced integration score; Liesefeld & Janczyk, 2019) as potential intermediate phenotype from baseline to 3-year follow-up. Application of ANCOVA (linear regression with dummy variable for baseline smoking vs. baseline no smoking and baseline GoNogo performance as covariate) with assumed correlation of 0.5 between baseline GoNogo and 3-year follow-up GoNogo and an assumed small-to-medium standardized effect size (equivalent Cohen’s d) of 0.3 for the GoNogo performance change difference between baseline smoking and baseline no smoking will yield a power of 86% if all formal statistical assumptions were met. These considerations yield a very rough reference point to expected statistical power for prospective analysis which obviously depends heavily on actual effect sizes and actual group sizes of specific analyses conducted. The true actual power will also vary according to many factors, e.g. the actual number and variances of covariates in the regression model and possible additional sample weighting procedures accounting for possible systematic dropout.

References:

Baltasar-Tello, I., Miguelez-Fernandez, C., Penuelas-Calvo, I., & Carballo, J. J. (2018). Ecological Momentary Assessment and Mood Disorders in Children and Adolescents: a Systematic Review. *Curr Psychiatry Rep, 20*(8), 66. doi:10.1007/s11920-018-0913-z

Demidenko, E. (2008). Sample size and optimal design for logistic regression with binary interaction. *Stat Med, 27*(1), 36-46. doi:10.1002/sim.2980

Ellis, B. J., Boyce, W. T., Belsky, J., Bakermans-Kranenburg, M. J., & van Ijzendoorn, M. H. (2011). Differential susceptibility to the environment: An evolutionary–neurodevelopmental theory. *Development and Psychopathology, 23*(1), 7-28. doi:10.1017/S0954579410000611

Heron, K. E., Everhart, R. S., McHale, S. M., & Smyth, J. M. (2017). Using Mobile-Technology-Based Ecological Momentary Assessment (EMA) Methods With Youth: A Systematic Review and Recommendations. *J Pediatr Psychol, 42*(10), 1087-1107. doi:10.1093/jpepsy/jsx078

Jones, A., Remmerswaal, D., Verveer, I., Robinson, E., Franken, I. H. A., Wen, C. K. F., & Field, M. (2019). Compliance with ecological momentary assessment protocols in substance users: a meta-analysis. *Addiction, 114*(4), 609-619. doi:10.1111/add.14503

Liesefeld, H. R., & Janczyk, M. (2019). Combining speed and accuracy to control for speed-accuracy trade-offs(?). *Behav Res Methods, 51*(1), 40-60. doi:10.3758/s13428-018-1076-x

Pluess, M. (2015). Individual differences in environmental sensitivity. *Child Development Perspectives, 9*(3), 138-143. doi:10.1111/cdep.12120

StataCorp. (2017). Stata statistical software: release 15. College Station, TX: Stata Corporation.

**Supplement B: Overview of completed BeMIND baseline assessments**
